# The Ebola outbreak, 2013–2016: old lessons for new epidemics

**DOI:** 10.1098/rstb.2016.0297

**Published:** 2017-04-10

**Authors:** Cordelia E. M. Coltart, Benjamin Lindsey, Isaac Ghinai, Anne M. Johnson, David L. Heymann

**Affiliations:** 1 Research Department of Infection and Population Health, UCL, London WC1E 6JB, UK; 2 Chatham House, London SW1Y 4LE, UK; 3 London School of Hygiene and Tropical Medicine, London WC1E 7HT, UK

**Keywords:** Ebola, Ebola virus disease, Ebola outbreak, West Africa

## Abstract

Ebola virus causes a severe haemorrhagic fever in humans with high case fatality and significant epidemic potential. The 2013–2016 outbreak in West Africa was unprecedented in scale, being larger than all previous outbreaks combined, with 28 646 reported cases and 11 323 reported deaths. It was also unique in its geographical distribution and multicountry spread. It is vital that the lessons learned from the world's largest Ebola outbreak are not lost. This article aims to provide a detailed description of the evolution of the outbreak. We contextualize this outbreak in relation to previous Ebola outbreaks and outline the theories regarding its origins and emergence. The outbreak is described by country, in chronological order, including epidemiological parameters and implementation of outbreak containment strategies. We then summarize the factors that led to rapid and extensive propagation, as well as highlight the key successes, failures and lessons learned from this outbreak and the response.

This article is part of the themed issue ‘The 2013–2016 West African Ebola epidemic: data, decision-making and disease control’.

## 1. Introduction

Ebola virus is a member of the Filoviridae family. There are five strains that have been identified: *Zaire*, *Sudan*, *Bundibugyo*, *Taï Forest* and *Reston*. The first three cause the majority of disease in humans with case fatality rates ranging from 25% to 90% [1–3]. Both *Taï Fores*t and *Reston* Ebola virus cause diseases in non-human primates, but infections in humans are limited to one case of *Taï Forest* Ebola virus and largely asymptomatic infections with *Reston* Ebola virus [4].

Ebola viruses have significant epidemic potential, as shown by the 2013–2016 West African outbreak. Caused by the *Zaire* strain, this outbreak was unprecedented in scale, being larger than all other outbreaks combined, with 28 646 reported cases (confirmed, probable and suspected) and 11 323 reported deaths [5]. This Ebola outbreak was the first to lead to a major global public health threat and the first in which the virus spread across multiple international boundaries.

Although Ebola is known to cause outbreaks in central and eastern Africa, no sporadic human cases or outbreaks had previously been reported in West Africa. Notwithstanding this, there was a major outbreak in 1994 among non-human primates in Cote d'Ivoire with one veterinarian subsequently infected. Because of this, in the early phase of the West African outbreak Ebola was not considered as a differential diagnosis, with Lassa fever—another viral haemorrhagic fever that is known to occur in humans in West Africa—being considered a more likely cause.

The widespread nature of the West African outbreak is thought to be related to the highly mobile communities and densely populated regions affected in the early stages of the outbreak. Previous outbreaks had been limited to remote, rural areas allowing initial containment efforts to be more effective. The majority of cases in the West African outbreak were localized to three countries with intense transmission: Sierra Leone, Liberia and Guinea. Seven other countries had minor outbreaks with non-sustained transmission or isolated cases, all with origins attributable to West Africa: Nigeria, Mali, Senegal, Spain, the UK, the USA and Italy. Other countries also accepted evacuated cases from West Africa for hospitalization, including Germany, France, Switzerland, the Netherlands and Norway.

This article details the evolution of the 2013–2016 West African outbreak and aims to summarize the events by country in chronological order. The landscape of previous Ebola outbreaks is described in order to highlight differences and we summarize theories about the origins of the 2013–2016 outbreak, as well as the factors that led to outbreak propagation. Finally, we review and discuss the key successes, failures and lessons learned from this outbreak.

## 2. Previous Ebola outbreaks

There have been 29 outbreaks or case reports of Ebola virus disease (EVD) reported since Ebola was first identified in 1976 (table 1). The majority of human disease has arisen from 15 outbreaks caused by the *Zaire* strain and eight by the *Sudan* strain. These outbreaks and cases were limited to rural communities in Sudan, Democratic Republic of Congo (DRC), Republic of Congo, Gabon and Uganda. Most of these outbreaks were small in size with just seven outbreaks involving more than 100 cases. The largest of the outbreaks before 2013 occurred in Uganda in 2000–2001 with 425 cases and 224 deaths (*Sudan* strain) and DRC (Kikwit) in 1995 with 315 cases and 250 deaths (*Zaire* strain) [6].

**Table 1.** Previous Ebola outbreaks/infections in humans. (Adapted from CDC [6].)

year	countries	no. outbreaks	no. cases	no. deaths	viral strain
1970–1979	Zaire, 1976^a^	2	319	281	*Zaire*
	Sudan, 1976^b^	2	318	173	*Sudan*
	United Kingdom, 1976	1	1	0	*Sudan*
1980–1989	Philippines, 1989–1990	1	3^c^	0	*Reston*
1990–1999	USA, 1990	1	4^c^	0	*Reston*
	Gabon, 1994	3	149	97	*Zaire*
	Côte d'Ivoire, 1994^d^	1	1	0	*Taï Forest*
	DRC, 1995	1	315	250	*Zaire*
	Gabon/South Africa, 1996^e^	1	2	1	*Zaire*
	Russia, 1996	1	1	1	*Zaire*
2000–2009	Uganda, 2000–2001	2	574	261	*Sudan/Bundibugyo*
	Gabon, 2001–2002	1	65	53	*Zaire*
	Republic of Congo, 2001–2002	3	235	200	*Zaire*
	Sudan^b^, 2004	1	17	7	*Sudan*
	Russia, 2004	1	1	1	*Zaire*
	DRC, 2007	2	296	202	*Zaire*
	Philippines, 2008	1	6^c^	0	*Reston*
2010–2013	Uganda, 2011–2013	3	18	8	*Sudan*
	DRC, 2012	1	36	13	*Bundibugyo*

^a^Now Democratic Republic of Congo (DRC).

^b^Now South Sudan.

^c^Asymptomatic infection.

^d^Patient was hospitalized in Basel, Switzerland for medical treatment.

^e^Index case infected in Gabon, admitted to hospital in South Africa for treatment and subsequently infected a nurse.

The first documented outbreak of Ebola occurred in 1976 in northern Zaire (now the Democratic Republic of Congo). It occurred in and around a mission hospital in Yambuku, adjacent to the Ebola River after which the virus was named. In total, 318 cases were identified with a case fatality of 88% [2]. Nosocomial transmission played a significant role in this first outbreak, as it would in future Ebola outbreaks, during which the reuse of contaminated needles in the local hospital was found to be the major factor in the initial spread of the virus.

Two months prior to the outbreak in Zaire in 1976 a similar outbreak occurred in the south of Sudan, which initially did not attract international attention [7]. The Sudan outbreak originated in a cotton factory in Nzara and was amplified by transmission in a local hospital in Maridi, both of which were close to the border with Zaire. Owing to the proximity of the subsequent outbreak in Zaire, these two outbreaks were initially thought to be linked. However, they were subsequently found to be caused by two different strains of Ebola virus later named *Zaire* and *Sudan* strains respectively. A total of 284 cases were identified in the Sudanese outbreak with a case fatality of 53% [8].

An additional strain, *Reston* Ebola virus, was first identified in the USA in monkeys imported from the Philippines in 1989. The virus was transmitted to humans working with the chimpanzees, as shown by an antibody response. Although none showed classical symptoms of EVD (box 1), mild fever and non-specific symptoms were reported. This strain has also been identified in pigs with asymptomatic transmission to at least six humans in contact with pigs reported in the Philippines [6].

Box 1. **Ebola virus disease—key facts about the virus, illness, management and prevention. (Adapted from CDC [9].)**

**• Outbreak emergence**

— EVD is a zoonotic infection—outbreaks begin in humans following a spillover event from an animal to human.
— The animal reservoir for Ebola remains uncertain, but current evidence suggests that bat species may be a natural reservoir, with other species acting as intermediate hosts, particularly non-human primates [10]. The evidence to support this includes:
  (i) Experimental inoculation of animals epidemiologically linked with outbreaks of Ebola virus demonstrated viral replication in fruit and insectivorous bats without fatalities [11]
  (ii) Trapping studies have found wild bats with evidence of Ebola infection and immunity (RNA fragments and Ebola-specific antibodies) in areas which have had human and non-human primate Ebola outbreaks [12]. However, live Ebola virus has never been isolated from a bat in nature, which makes the picture less clear [13]
  (iii) Ebola virus has been isolated from other animals, including non-human primates and duikers, but these species appear to be unsuitable reservoirs because they have high case fatality rates during outbreaks [10,13].
— Bats have been suggested as a possible source for the 2013–2016 epidemic, based on retrospective epidemiological data:
  (i) Interviews with contacts of the index case in Guinea indicated that the two-year old child regularly played near a tree found to house a colony of insectivorous bats [14].
  (ii) The species of bats known to carry Ebola virus were identified close to where the index case lived.
  (iii) No evidence of Ebola virus infection was found in specimens obtained from bats in this area based on RT-PCR and serology assays (*n*=169 specimens) [14].

**• Disease transmission**

— Outbreaks of EVD are thought to originate when the index case becomes infected through contact with the body fluids of an infected animal. Once the index case becomes ill or dies, the virus spreads to others who come into direct contact with the blood or body fluids of the infected person.
— Individuals are only considered to be infectious when symptomatic (not when asymptomatic during the incubation period).
— Multiple estimates of the basic reproductive number, R0, have been carried out for the 2013–2016 epidemic. R0 is defined as the number of secondary cases that arise from a primary case in a completely susceptible population. In Guinea, estimates ranged from 1.5 to 1.71, compared to 1.36 and 1.83 in Liberia, and estimates ranged more widely from 1.4 to 2.53 in Sierra Leone [15].

**• Clinical presentation**

— Incubation period: 2-21 days (mean 11.4 days) between infection and symptom onset during this 2013-2016 outbreak [16]. This is similar to previous reports.
— Symptoms:
  (i) Most cases of EVD begin with the abrupt onset of fever and malaise.
  (ii) There is a broad spectrum of symptom presentation from very mild to severe haemorrhagic complications (rare).
  (iii) Common early symptoms include headache, vomiting, diarrhoea, myalgia, rash, and hiccups, and can then lead to haemorrhagic symptoms, uveitis, conjunctival injection and neurological symptoms (meningoencephalitis with altered consciousness, neck stiffness, and seizures).
  (iv) Gastrointestinal symptoms are very common and can lead to significant fluid loss and electrolyte disturbance leading to hypovolaemic shock or arrhythmias and sudden death.
  (v) Traditionally, haemorrhagic symptoms were thought to be the defining feature and indeed EVD was previously named ‘Ebola haemorrhagic fever’, but major haemorrhages are not seen in the majority of patients, although a degree of bleeding (blood in stool, petechiae, ecchymoses, oozing from venepuncture sites, and mucosal bleeding) can occur. Significant bleeding is seen in the terminal phase of some cases and in pregnancy.
— Most patients who survive EVD show signs of improvement during the second week of illness.

**• Investigations**

— Testing for the presence of Ebola virus is by reverse-transcription polymerase chain reaction (RT-PCR).
— Other laboratory findings associated with EVD include leukopenia, thrombocytopenia, serum transaminase elevations (secondary to multifocal hepatic necrosis), decreased serum albumin, elevated amylase, electrolyte disturbances, renal and coagulation abnormalities.
— Testing to exclude other differential diagnoses of febrile illnesses should also be undertaken. This is particularly relevant for patients who are PCR-negative, but should be done, where possible in all cases, to exclude comorbidity; malaria coinfection, for example, is not uncommon.

**• Treatment**

— Early diagnosis is essential in order to initiate treatment and institute strict infection control.
— There are no approved therapies and management is largely supportive. Maintaining fluid balance with electrolyte replenishment, empirical antibiotics and anti-diarrhoeal agents are important. The use of non-steroidal anti-inflammatory drugs and vitamin K remains contentious. Different protocols exist across different settings and organizations.
— Despite no licensed treatment options, significant progress was made during the outbreak with 15 experimental therapies trialled on humans. These include blood products e.g. convalescent plasma, immunological agents (e.g. monoclonal antibodies (e.g. ZMapp)), and antiviral drug therapies (e.g. Favipiravir and novel agents (TKM0120803)) [17]. In addition, the efficacy of vaccine candidates was also studied.

**• Preventative strategies**

Strategies to prevent transmission of Ebola virus during an outbreak include:

— Infection control (including patient isolation) and sterilization including careful hand hygiene (wash hands with soap and water or an alcohol-based sanitizer), avoidance of contact with blood and body fluids of Ebola patients (e.g. urine, faeces, saliva, sweat, urine, vomit, breast milk, semen, and vaginal fluids), and avoidance of all items in contact with infected patients (e.g. bedding, needles/medical equipment)
— Use of personal protective equipment where appropriate
— Contact tracing and fever surveillance of all contacts with quarantine where required
— Safe burial - avoiding rituals that require washing or handling of the body of an Ebola patient
— Safe sex advice
— Engaging communities in preventing transmission
— Vaccination strategies (although no vaccine is currently licensed and this would not prevent random emergence events).

In 1994, the *Taï Forest* Ebola virus was reported to cause an outbreak among chimpanzees in Cote d'Ivoire. A veterinarian studying the outbreak was subsequently infected following post-mortem examinations on the infected chimpanzees [18]. She is the only known human to have been infected with this strain and survived after being hospitalized in Basel, Switzerland.

The *Bundibugyo* strain was responsible for two outbreaks in Uganda in 2007 and Democratic Republic of Congo in 2012. The Ugandan outbreak resulted in 131 cases and appears to have the lowest mortality (32%) of all viral strains that cause EVD in humans [6].

In August 2014, during the height of West African outbreak, a case of EVD was confirmed in rural Jeera County, DRC (*Zaire* strain). Sequencing data showed the two outbreaks to be unrelated, with the index case identified as a pregnant woman infected after contact with bush meat. The scale of this outbreak was in keeping with pre-2013 outbreaks, with 66 reported cases and 49 deaths [19].

In addition to the outbreaks described here, isolated individual human cases have been reported including imported cases from Gabon to Johannesburg, South Africa (*Zaire* strain) and three laboratory accidents—one in the United Kingdom (*Sudan* strain) and two in Russia (*Zaire* strain).

Of note is the discovery that, although there are no previous reports of EVD outbreaks in West Africa, retrospective testing of archived clinical samples during the 2013–2016 outbreak revealed EVD was causing clinical symptoms in Sierra Leone as early as 2006 [20].

## 3. Ebola virus disease: clinical picture

While the focus of this article is not the clinical presentation and management of persons with Ebola virus infection, we highlight the key details of EVD in box 1 in order to help contextualize the infection and outbreak response. This includes a summary of clinical presentation as well as strategies for diagnosis, management and prevention.

## 4. Outbreak evolution

**Figure 1.**
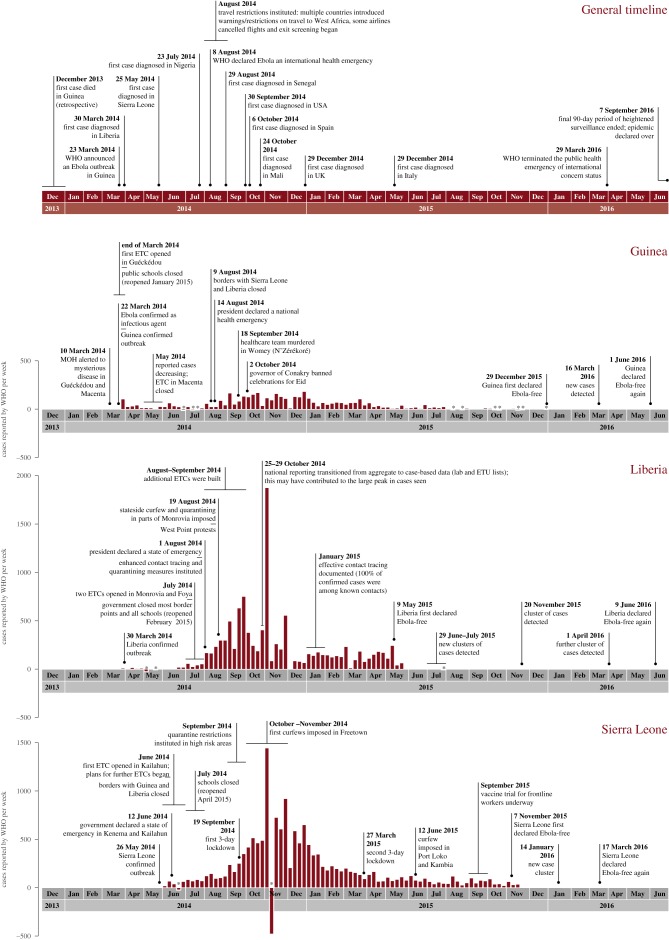
Timeline of key events with country-specific epidemic curves. Case numbers are total reported confirmed, probable, and suspected cases provided in WHO situation reports throughout the epidemic. We have calculated weekly case number (Monday–Sunday). Asterisk indicates where negative case numbers are reported (as per WHO data) when suspected/probable cases subsequently test negative for Ebola PCR or as a result of data errors. MOH, Ministry of Health; MSF, Médecins Sans Frontières; ETCs, Ebola treatment centres. Where events happened multiple times, only the first occurrence has been shown.

**Figure 2.**
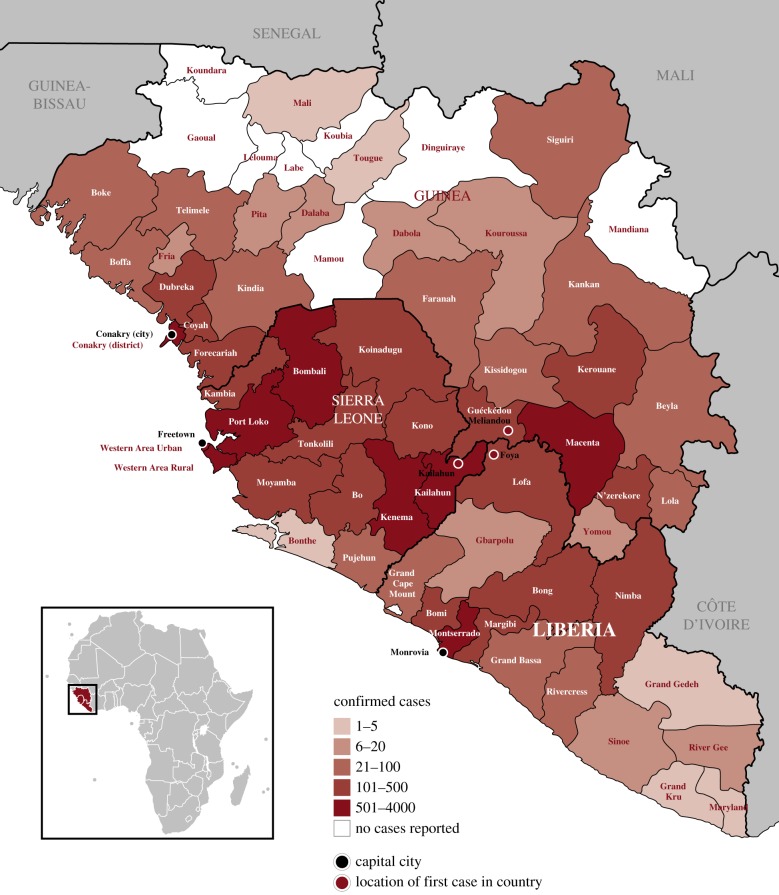
Geographical map of Guinea, Sierra Leone and Liberia showing districts and total number of confirmed cases by district. (Adapted from WHO [21].)

The outbreak began in Guinea with the first case retrospectively identified as having occurred in late 2013 and spread to several other countries, with Sierra Leone and Liberia most severely affected. The evolution of the 2013–2016 West African outbreak is described below in chronological order, by country. Figure 1 documents a timeline of the major outbreak events in each of the three main affected countries, including epidemic curves. Figure 2 shows a map of the three countries in West Africa with intense transmission, to help visualize the geographical spread outlined. Table 2 shows a summary of basic country statistics pre-Ebola. Box 2 highlights key outbreak-related definitions used throughout the paper.

**Table 2.** Basic country statistics from the three main affected countries. (Adapted from World Bank data, 2014, unless otherwise stated [22,23].)

country statistic	Guinea	Liberia	Sierra Leone
population	12.3 million	4.4 million	6.3 million
rural population (% of total)	63.3	50.7	60.4
gross domestic product *per capita* (US$)	539.6	457.9	792.6
capital city	Conakry	Monrovia	Freetown
physicians per 1000 people (as of 2010)	0.1	0.014	0.022
total number of reported Ebola cases^†^ (WHO 2013–2016)	3811	10 678	14 124
total number of Ebola deaths (WHO 2013–2016)	2543	4810	3956

^†^confirmed, probable, and suspected cases reported by WHO.

### (a) Guinea

On 10 March 2014, the Ministry of Health in Guinea was alerted to an outbreak of a mysterious disease characterized by fever, severe diarrhoea, vomiting and a high fatality rate in the prefectures (regions) of Guéckédou and Macenta in southeast Guinea (figure 2).^1^ Two days later, Médecin Sans Frontières (MSF), which had worked in the region primarily on malaria projects since 2010, was also notified. A team sent by the Ministry of Health reached the outbreak area on 14 March, with a European MSF team arriving on 18 March. Epidemiological investigation and blood samples (sent to the biosafety level 4 laboratories in Lyon, France and Hamburg, Germany) confirmed EVD. The WHO subsequently announced an Ebola outbreak on 23 March 2014 [26–28].

Box 2. **Outbreak-related definitions used.**

**• WHO case definitions used during an outbreak [24]**

Suspected case

Any person, alive or dead, suffering or having suffered from a sudden onset of high fever and having had contact with:

— a suspected, probable or confirmed Ebola case,
— a dead or sick animal.
Any person with sudden onset of high fever and at least three of the following:
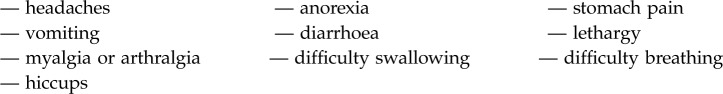

Any person with inexplicable bleeding;

Or any sudden, inexplicable death.

Probable case

Any suspected case evaluated by a clinician; any deceased suspected case having an epidemiological link with a confirmed case (where it is not possible to collect samples for laboratory confirmation).

Confirmed case

Laboratory-confirmed cases: any suspected or probable cases testing positive for the virus (virus RNA by reverse transcriptase-polymerase chain reaction (RT-PCR) or IgM antibodies against Ebola).

N.B. In this paper the use of ‘case’ or ‘case number’ refers to the total reported confirmed, probable, and suspected cases provided in WHO situation reports throughout the epidemic.

**• WHO criteria for declaring the end of the Ebola outbreak [25]**

End of Ebola outbreak: 42 day count

The outbreak is considered ended in a country after 42 days have passed since the last confirmed case has met one of three criteria:

— been isolated, recovered and subsequently tested negative for the virus on two blood samples (collected at an interval of at least 48 hours)
— been isolated and subsequently died in an ETC with a safe burial organized by the ETC. The 42 day count begins on the day following burial
— the case was a contact of a confirmed Ebola case. He/she died and was buried in the community and was either a confirmed case or a probable case. The 42 day count begins on the day following burial.

The outbreak in the West Africa subregion was declared over when the 42 day period elapsed in the last affected country.

The rationale for 42 days is based on twice the maximum incubation period for Ebola, as this can be expected to confirm the interruption of human-to-human transmission chains.

During the 42 day period each country should:

— ensure active case finding around confirmed cases and transmission chains
— implement both active and passive surveillance for EVD (e.g. through regular health facility visits and by maintaining a nationwide system of alerts and signals)
— conduct post-mortem testing for EVD following deaths in the community
— offer testing of semen samples among survivors and, for those who test positive, monthly testing thereafter until two negative results are obtained
— ensure screening of blood donors and products
— sentinel surveillance among patients with febrile illness.

90 day heightened surveillance period

After the 42 day period has elapsed, each country should maintain a system of heightened surveillance for a further 90 days, ensuring ongoing EVD surveillance and notification. During this time a combination of active and passive surveillance should be maintained, ideally integrated with surveillance for other important epidemic-prone diseases. Post-mortem testing and testing of survivor semen samples should also be continued for 90 days. Passive surveillance should then be continued indefinitely and EVD preparedness plans should be in place and monitored in all countries previously affected by EVD.

The rationale for 90 days of heightened surveillance is due to:

— the continued risk of new importations of EVD within the West Africa sub-region, given the outbreak occurred in multiple countries
— the possibility of sexual transmission (current evidence suggest that viable Ebola virus can persist in semen for at least 82 days after symptom onset and possibly longer than six months)
— the possibility of a missed transmission chain
— the possibility of a new emergence event from an animal reservoir.

**Figure 3.**
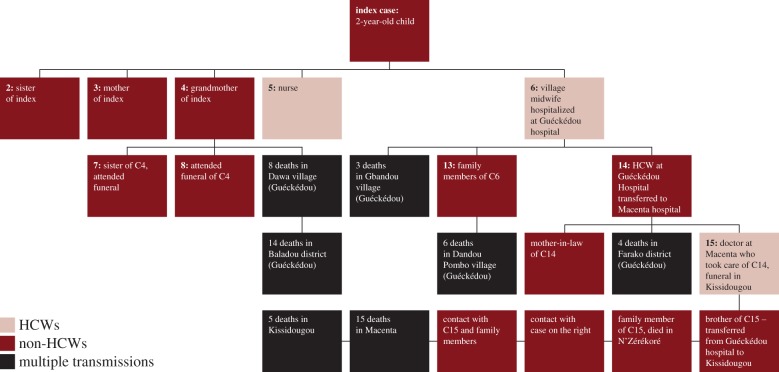
Initial transmission chain in Guinea. HCW, healthcare worker. (Adapted from Baize *et al.* [29] and Coltart *et al.* [30].)

Retrospective analyses traced the source of the outbreak to Meliandou village within the prefecture of Guéckédou, in the forested region of southeastern Guinea that borders both Sierra Leone and Liberia. The suspected index case was a two-year old child who fell ill on 2 December 2013 and died 4 days later [29]. A second epidemiological investigation confirmed the source village and index case, but the date of death of the index case was documented as the end of December 2013. Other family members rapidly became unwell and died between 13 December 2013 and 1 January 2014 (the index case's mother, sister, and grandmother). A village midwife who cared for the index case during his illness also fell ill—during her hospitalization in the nearest town, Guéckédou, she likely infected another healthcare worker (HCW) who was hospitalized in Macenta Hospital and is thought to have triggered the spread of the infection to a larger town. The midwife also had epidemiological links to cases in villages around Guéckédou prefecture (Dandou Pombo, Dawa and Gbandou Villages) between January and March 2014. The initial case fatality rate was 86% (12 of the 14 original patients with a known outcome died). Baize *et al.* reported this initial transmission chain in October 2014, and an adaptation of the initial transmission tree is shown in figure 3.

Between December 2013 and the identification of Ebola virus as the infectious agent at the end of March 2014, a total of 49 cases met the case definition for EVD, with 111 clinically suspected cases and 79 deaths attributed to EVD on the basis of clinical symptoms. The cases were recorded in the prefectures of Guéckédou, Macenta and Kissidougou.

The first cases in the capital city, Conakry, were detected in March 2014. However, it was not until May 2014 that sustained transmission in Conakry was documented. Conakry is a city of approximately two million inhabitants located in the west of the country on the coast, approximately 400 km from Meliandou [27]. While most districts had a peak or peaks of infection, both Guéckédou and Conakry had fairly consistent levels of transmission spanning the majority of the outbreak period in Guinea.

By May 2014, the number of cases appeared to be declining in the initial epicentre leading to an MSF treatment facility in Macenta being closed. New cases continued to be reported in other parts of the country, but these were attributed to introductions from Sierra Leone and Liberia. In time it became apparent that case numbers had not fallen; instead cases had been hidden owing to a number of conspiracy theories which arose when patients were taken to Ebola treatment centres (ETCs) and died there. In September 2014, one newspaper reported that ‘many Guineans say local and foreign HCWs are part of a conspiracy which either deliberately introduced the outbreak, or invented it as a means of luring Africans to clinics to harvest their blood and organs’ [31]. Furthermore, traditional burial practices were forbidden to reduce exposure to infected bodies (box 3). However, this violation of cultural beliefs bred fear that the deceased and their relatives would be cursed for failing to perform a proper ceremony. The unfamiliar sight of workers wearing full protective clothing heightened tensions; fear reached a climax when riots broke out in N'Zérékoré after rumours spread that medics disinfecting a market were in fact contaminating people [34]. Tragically, in September 2014 eight members of a healthcare team were murdered by residents of Womey near N'Zérékoré [34–36].

Box 3. **Key facts about traditional burial practices—why do we need safe burials? (Adapted from WHO and National Geographic [32,33].)**

• **Purpose of burial rituals**

The purpose of the burial rituals are threefold:

1. to honour the dead relatives in the traditional way
2. to say good-bye
3. to accommodate the deeply held beliefs about the obligations of the living to the dying and dead, respecting the cultural view of life after death.

• **Ceremony rituals**

— Many tribal ceremony rituals are closely held secrets, therefore, they are not well documented. However, one of the main rituals common to all groups is the washing of (with bare hands) and spending time with the dead body, which is highly infectious in the case of Ebola. Both of these traditions lead to enhanced transmission of infection and thus, highlight the need for safe burial practices.
— Burial ceremony traditions depend on tribe and religion. The populations affected by the Ebola outbreak consisted mainly of Christians and Muslims:
  (i) Christians close the eyelids, wash and dress the deceased
  (ii) Muslims wash the dead as well, then wrap the deceased in a white cloth
— Special circumstances:
  (i) Tribal leaders: additional rituals are undertaken to transfer powers to a successor
  (ii) Pregnancy: many cultural groups feel that the fetus needs to be removed from the mother's body before burial to prevent disturbing the world's natural cycles
  (iii) Some tribal rituals involve animal sacrifice, or inspecting the dead body to determine if the deceased had been a witch—if so, the spirits must be rendered innocuous before burial
— If not undertaken properly, there are consequences for both the deceased and the living relatives, for example the dead are thought to wander the earth eternally and plague the community if they do not reach the village of the dead.

• **WHO protocol for the management of a safe and dignified burial includes:**

— always take into account cultural and religious concerns and obtain family consent in burial plans
— only trained personnel should handle remains during the outbreak
— use Personal Protective Equipment (PPE)
— place the body in the body bag
— place the body bag in a coffin where culturally appropriate
— sanitize family's environment
— remove PPE, manage waste and perform hand hygiene
— transport the coffin or the body bag to the cemetery
— burial at the cemetery: place coffin or body bag into the grave
— engaging community for prayers as this dissipates tensions.

• **Challenges to implementing safe burial**

One of the main challenges in implementing safe burial practices during this epidemic was finding culturally acceptable methods in accordance with safety procedures. Genuine and open collaboration was required between political, health, tribal and religious leaders. WHO published the new protocols for safe and dignified burials in October 2014, designed in conjunction with affected communities, recognizing the need for the family and religious engagement. Examples of adaptations to burial practices used in the Ebola outbreak include:

— For deceased Muslims, shrouding the body in white body bags rather than the traditional white cotton [33]
— Guidance given by religious leaders suggesting alterations to traditional rituals, e.g.
  (i) not to wash the corpse, with some suggesting dry ablutions (performed in PPE) instead
  (ii) praying for the deceased in absentia was sufficient—this was believed to be acceptable as it occurred in historical outbreaks documented in the scriptures.
— Burial workers would try to honour reasonable requests from families of the deceased, e.g.
  (i) burial workers dressed in protective suits were permitted to dress the dead in outfits chosen by family before the corpses were placed in body bags
  (ii) money and jewellery or other sentimental items were allowed to be placed in body bags with the corpse as a ‘toll’ as some traditions believe the deceased must pay to cross over to the village of the dead
  (iii) brief prayers were allowed for loved ones, either while standing two meters (6.5 feet) from the white body bag before removal for burial, or at the grave site after burial.

Data during this early phase of the outbreak were irregularly collected and resources were focused on providing clinical care. Despite multiple requests from MSF and other agencies working in the country, it was not until 8 August 2014 that the WHO declared the outbreak a public health emergency of international concern. The President of Guinea, Alpha Conde, followed suit, declaring a national health emergency. Containment efforts included automatic admission to hospital for suspected cases, compulsory quarantining of Ebola contacts, travel restrictions including enhanced border controls, and preventing dead bodies being transported between towns (common for cultural repatriation). Contravening these restrictions would be subject to law enforcements [37].

Despite the increased international support that followed these pronouncements, weekly confirmed case numbers remained stubbornly between 75 and 148 between September and December 2014 [5]. In early October MSF reported a spike in cases in the capital, Conakry, with one treatment centre receiving 22 patients in a single day. As a result, the governor of Conakry banned all cultural celebrations for Eid [38,39].

During October, districts which were previously disease-free started reporting cases including Lola, Kankan and Faranah districts [5]. Transmission in Kankan and Faranah districts was of particular concern owing to their proximity to national borders; Kankan is adjacent to Côte d'Ivoire and forms part of the major trade route to Mali, whereas Faranah district neighbours Koinadugu in Sierra Leone, which was also beginning to report cases. The potential cross-border transmission highlighted the need for national border surveillance.

On 23 October 2014, the government of Guinea announced that they had started compensating the families of HCWs who died of EVD with a lump sum payment of $10 000 to each family. Prior to that date, 42 cases of HCW occupation-related death had been reported [40].

Intense transmission persisted through November and December. Concerns were raised on 20 November 2014 when the Red Cross sent blood samples to a testing centre via courier taxi. The taxi was robbed near the town of Kissidougou with the robbers unwittingly stealing the cooler bag with the infected blood. Despite public appeals, the samples were never recovered [41].

By mid-December 2014, cases were reported in the northern district of Siguiri bordering Mali [5] and, one month later, the virus had spread to the western district of Fria for the first time. In all, 19 districts were reporting transmission events on a weekly basis and transmission in the capital remained high [5].

All countries struggled to provide the necessary bed capacity to isolate and treat all confirmed, probable, and suspected cases of Ebola, particularly in the early phases of the outbreak. In total, Guinea had nine ETCs during the outbreak. The first ETCs to be established were run by MSF and the French Red Cross, with many other organizations forming crucial collaborations and providing resources and staff. The largest ETC was the first one in Guéckédou, which had a maximal capacity of 170 beds. Other centres included Macenta, Conakry (Donka Hospital), Coyah, Beyla, N'Zérékoré, Kerouane, Wonkifong, and Kankan [42]. Setting up an ETC was a huge undertaking and took time, meaning that demand rarely coincided with supply.

The number of new reported infections began to fall in early January 2015 to approximately 50 cases per week, although this continued to fluctuate [5]. Throughout February and March transmission was concentrated in the western districts including Conakry, Coyah, and Forecariah. The latter borders Kambia in Sierra Leone which was, by that time, in the midst of its own outbreak (see below). These last two districts (named above) were alone responsible for 46% of the weekly new case counts across all three West African countries towards the end of March 2015 [5].

During April–June 2015, the reported country weekly case count declined to approximately 20 cases per week, and fluctuated around this level until a further decrease at the end of July. Transmission remained concentrated in and around the western districts of Conakry and Forecariah, but with cases re-emerging elsewhere, for example in Dubreka and Boke. During August, Guinea reported only a few cases per week, focused around Conakry and Coyah; and 13 September 2015 marked the end of the first week during which no new cases were documented. However, a small number of additional intermittent cases were reported in the Forcariah district between mid-September and late October [5].

Guinea was first declared Ebola-free on 29 December 2015 after a 42 day period without new cases [5]. However, on 16 March 2016, within the 90 day high-level surveillance period, Guinean health officials reported three deaths with symptoms consistent with Ebola in the village of Koropara (N'Zérékoré prefecture) [43,44]. Investigators from the Ministry of Health, WHO, the US Centres for Disease Control (CDC), and UNICEF arrived the following day. Samples were taken from four contacts of the deceased and two tested positive for Ebola (the mother and sister of one of the deceased). Cases were rapidly admitted to a treatment facility together with rapid mobilization of epidemiologists, surveillance experts, vaccinators, social mobilizers, contact tracers and an anthropologist as part of the interagency response team. Guinea was again declared Ebola-free on 1 June 2016 [5].

Despite ultimately having the lowest confirmed number of cases and deaths of the three West African countries with major outbreaks, Guinea witnessed 2543 Ebola deaths among 3811 cases (confirmed, probable, and suspected), 3351 of which were laboratory-confirmed cases [5]. Of the 34 districts in Guinea, 24 were affected by Ebola, in contrast to both Liberia and Sierra Leone in which every district reported cases [21].

### (b) Liberia

The first cases of EVD were confirmed in Liberia on 30 March 2014, eight days after the outbreak was declared in Guinea: two cases confirmed in Foya district, Lofa County, close to the border with Guinea and Sierra Leone (figure 2) [45] with RNA sequencing confirming that the virus was imported from neighbouring Guinea [46].

The Liberian government responded to the outbreak by forming a high-level National Task Force composed of the WHO, UNICEF and multiple international non-governmental organizations (NGOs).^2^ Shortly after the formation of this task force the CDC sent teams to assist the response [27]. The initial response included enhanced surveillance, contact tracing, training of medical staff, community awareness campaigns, and supplying personal protective equipment to health facilities [45].

Within two weeks, five other counties had reported suspected cases: Margibi County, Bong, Nimba, Grand Cape Mount, and Montserrado [27]. Within the first month of the outbreak in Liberia 13 cases were confirmed, of which 11 died [47]. The initial wave of infection was effectively contained within Liberia with no new cases reported for nine weeks between 6 April and 7 June [27]. Phylogenetic analysis supports this, with no future samples of Ebola virus identified from the lineage responsible for the first wave of the outbreak [48].

Believing the outbreak to be ‘relatively small’ [49], the WHO and CDC began to withdraw from Liberia [50] despite ongoing transmission in neighbouring Guinea and calls from MSF that more support was required owing to the unprecedented geographical spread of the outbreak [51]. This withdrawal later formed part of the wider criticism of the international response to the outbreak [52].

A new laboratory-confirmed case of EVD was reported on 7 June 2014 in Foya district sparking the beginning of the second wave of transmission in Liberia [27]. Evidence from contact tracing and phylogenetic analysis suggests that the virus was reintroduced from Sierra Leone as it was a distinct viral lineage to previous cases in Liberia [48]. Genetic sequencing suggests that it was this second distinct viral lineage that was responsible for future intra-country spread, not the lineage responsible for the first importation in March 2014 [48]. The outbreak soon spread to Monrovia, the capital city where a quarter of the country's population live, and claimed the lives of a nurse and the head surgeon from Redemption Hospital, a reminder of the significant risk posed to HCWs during Ebola outbreaks [53,54]. By the end of June 2014 107 suspected cases of EVD had been reported in Liberia, including 52 laboratory-confirmed, with 65 deaths (in Lofa, Montserrado and Margibi).

In July 2014, two ETCs opened in Monrovia and Foya, run by the charity Samaritan's Purse. Capacity was minimal and centres filled quickly, the centre in Monrovia having only 40 beds [51]. Within a month two American volunteers were infected with Ebola—Samaritan's Purse subsequently suspended activities in the country and evacuated its staff, with MSF stepping in to manage the ETCs [51]. July also saw the Liberian Government close most border points and all schools in order to minimize transmission [55]. Despite this, by the end of July, case numbers had tripled and EVD had spread to seven of Liberia's 15 counties (including Lofa, Montserrado, Margibi, Bomi, Bong, Nimba, and Grand Gedeh); there were 329 suspected cases and 156 deaths [27].

Transmission increased rapidly throughout August and September 2014 (total cases 1082 and 3458, respectively), and all but two counties reported cases. Additional ETCs, run by MSF, Save the Children, International Medical Corps, and the Liberian Ministry of Health (MOH), were built but struggled to cope with the high volume of cases [56]. The 120 bed ‘Island Clinic’ in Monrovia, built by the Liberian MOH, reached capacity within 24 hours [57]. By early September, the total bed capacity in Liberia was 314, with an estimated deficit of 760 beds in Monrovia alone [5].

October 2014 saw the largest number of new cases in a month, with 3077 cases suspected or confirmed, nearly doubling previous case counts [47]. All 15 counties had now reported at least one confirmed case, with Montserrado, Magibi, Bong and Nimba worst affected, while transmission was decreasing in Foya [5].

In November 2014, the U.S. government proposed to send $2.89 billion and deploy 3000 troops to West Africa, with their response focused on Liberia [58,59]. This resulted in the building of new ETCs, an increase in laboratory capacity, air transport of supplies, and enhanced awareness programmes. However, Ebola virus transmission decreased significantly before many of the ETCs had become operational, with some ETCs treating no patients [59].

Mistrust between communities and authorities was a common theme in all countries affected by the West African outbreak. This was epitomized by protests in Liberia in mid-August 2014. Residents of the West Point District tried to dismantle an Ebola screening unit which they viewed as a risk to their safety. This lead to violent clashes between soldiers and protesters and the eventual quarantining of the whole West Point District [60]. Mistrust of the government, as well as fear of stigmatization, led some to avoid seeking medical help for suspected EVD and reluctance to engage in surveillance and contact tracing [56]. In a heavy-handed response, the Liberian Government made it illegal to conceal an Ebola-infected patient, punishable by a prison sentence of 2 years [61].

The peak in reported cases occurred in September 2014, but by late 2014 transmission had begun to decrease. By early January 2015, nine months after the first reported cases in Liberia, approximately 150 new cases were being reported per week, with transmission limited to two counties: Montserrado and Grand Cape Mount [47]. Effective contact tracing and monitoring were now well established; all registered contacts were being monitored daily, and 100% of new confirmed cases were occurring among known contacts [5].

The first week of March 2015 was the first week with no new reported cases of EVD in Liberia. One additional case was confirmed in Monrovia later in March but led to no additional subsequent infections. Forty-two days later, on 9 May 2015, Liberia was declared Ebola-free [5].

Liberia was declared Ebola-free three more times, after small clusters of infection in June 2015, November 2015 and April 2016. The first of these clusters was of six confirmed cases near Monrovia. Genomic sequencing suggested re-emergence from an EVD survivor in Liberia rather than cross-border spread [5]. The second cluster occurred in Monrovia among three members of the same family. This was also attributed to long-term viral carriage in a survivor. The final cluster occurred in April 2016 and was thought to have been imported from Guinea when a woman travelled to Monrovia from Macenta, Guinea, to visit relatives after the death of her husband from EVD. The virus spread to her two sons, but with rapid diagnosis, contact tracing, early treatment and isolation in an ETC, the virus did not spread further [62]. Liberia was again declared Ebola-free on 9 June 2016. The total number of confirmed, probable, and suspected cases in Liberia was 10 678 (3151 laboratory-confirmed), with 4810 deaths [47].

### (c) Sierra Leone

EVD was first confirmed in Sierra Leone on 25 May 2014 [63]. The first case was a young woman in Kenema, Sierra Leone's third largest city, 50 km from the Liberian border and 100 km from the border with Guinea. Given the situation in neighbouring countries, Sierra Leone had already begun an enhanced surveillance programme based in the Lassa fever isolation ward in Kenema General Hospital [64]. Within a month of the outbreak being confirmed in Sierra Leone, over 150 people were reported infected [47] and case numbers appeared to be increasing rapidly. The government declared a state of emergency in the ‘eastern hub’ of Kenema and neighbouring Kailahun on 12 June 2014 and WHO reinforced its representation in-country from mid-June [65]. Within six months of the first reported case, the outbreak in Sierra Leone peaked (November 2014) with up to 150 people a week being infected [47].

The steep gradient of the early part of the epidemic curve hints at a ‘slow and silent’ undetected early phase to the outbreak in Sierra Leone [66]. Retrospective analyses suggest that the Ebola virus was introduced to Sierra Leone from Guinea more than five months before the first officially reported case. This analysis identified a female who travelled from Meliandou, Guinea to Sierra Leone and subsequently died from EVD in January 2014 [66]. Further genomic analysis suggests two distinct linages of Ebola virus were introduced into Sierra Leone from Guinea in early 2014 [64].

MSF opened the first ETC in Sierra Leone in Kailahun in mid-June with diagnostic support from Public Health Canada. Like many ETCs at this stage of the outbreak, this facility was rapidly overwhelmed. Further beds were provided in the Lassa fever isolation ward at Kenema District Hospital, which was uniquely placed to deal with the emerging threat of EBV. However, this too was overwhelmed by the sheer number of cases and it was forced to move patients into general medical wards where isolation and infection control were inadequate. More than 40 HCWs from this hospital were infected in 2014 [67], with many of them dying, including Sierra Leone's only national expert on haemorrhagic fevers [68].

By mid-2014, the UK Government took an active role in supporting the National Ebola Response in Sierra Leone. Together with NGOs such as Save the Children, it provided four ETCs with 700 beds in major urban centres, including one specifically for HCWs infected with EVD, which was led by the British military [69].

Once the virus was established within Sierra Leone, molecular epidemiological evidence suggests that sustained human-to-human transmission occurred within the country, rather than through repeated cross-border reinfections or recurrent zoonotic events [70]. Ebola virus appears to have spread long distances following major roads networks, whereas many smaller chains of transmission went unnoted and uncontrolled in remote, isolated villages [71]. Just as the outbreak in one area was thought to be coming under control, these undetected links surfaced in new geographical areas, causing wave-like spread across the country from east to west.

By September 2014, sustained transmission was reported in the densely populated capital, Freetown [72]. The spread from the eastern hub to Freetown, which resulted in intense transmission, marked a serious escalation in the outbreak [73]. The government of Sierra Leone, out of ‘a desperate need to step up [their] response’ [74], began a range of measures aimed at containing the outbreak. A state of emergency was declared and a 3 day national lockdown was imposed in September 2014. The lockdown was designed not only to decrease the movement of people, but also to give HCWs time to identify new cases and increase awareness of EVD through door-to-door campaigns [75]. Subsequently, quarantine restrictions were put in place in high-risk areas; curfews were imposed, including in Freetown, lasting anywhere from 21 days to several months, with restriction of movements between 18.00 and 06.00 daily; schools and other public places were closed; all large gatherings including sporting events were cancelled [76]; and screening at land borders was strengthened [66].

Mass quarantine proved controversial—at one point one third of the population of Sierra Leone was under quarantine [77]. The president acknowledged the situation as ‘difficult’ [78], although this significantly underplays the nature of the food shortages that affected some of the quarantined areas and forced people to break quarantine [79]. Aside from the ethical concerns, many felt that mass quarantine measures were ineffective for Ebola as patients are not infectious until they become symptomatic [80], and they may have been counterproductive by preventing the free movement of necessary medical supplies and personnel [81].

By October 2014, transmission had spread to the northern district of Koinadugu, the last remaining Ebola-free district of Sierra Leone [82]. However, by November 2014, the outbreak in the eastern hub had begun to see reduced transmission [73].

More HCWs were infected and died in Sierra Leone than in any other country, both in absolute numbers and relative (proportion of cases) terms [67]. Sierra Leone was the only country in which there were strikes by frontline workers because of working conditions and pay. In late 2014, burial workers went on strike over unsafe working conditions and a lack of hazard pay [83], in contrast to the relatively generous compensation paid to HCWs and their families in Guinea [40]. In addition, doctors and nurses withdrew their labour [66] seeking assurance that a new UK-built treatment centre for HCWs would accept local, as well as international, staff if they became infected [84].

Despite an apparently well-functioning contact tracing system [85], cases continued in the north of the country across the first half of 2015, in part fuelled by secret and unsafe burials [86]. In June 2015, ‘Operation Northern Push’ was launched by the government of Sierra Leone, in collaboration with international partners, to eliminate Ebola from Port Loko and Kambia districts which were hotspots of transmission [87]. This included the imposition of curfews in both districts (12 June 2015), enhanced surveillance, active contact tracing, intense community engagement and mass quarantine. With these intense response strategies, the outbreak in Sierra Leone appeared to be coming under control, but despite the strictness of Operation Northern Push [88] new chains of transmission proved stubbornly resistant to detection. News of a case in Kambia in September 2015—three weeks after the last reported case and with no link to any known chain of transmission—led to a flurry of activity, including the first trial of ring vaccination in Sierra Leone for Ebola virus [89]. This was in addition to vaccine trials for frontline workers undertaken in Sierra Leone [90].

After this concerted effort, in November 2015, one year on from the epidemic peak, Sierra Leone was declared free of Ebola virus [91]. However, two months later in January 2016, a 22-year old woman died of EVD and her carer was subsequently found to be infected. Applying many of the lessons from the previous 18 months, the public health system responded rapidly and effectively, quickly containing the flare-up and preventing spread. Four months later, and almost 2 years after the first confirmed infection, Sierra Leone was again declared Ebola-free on 17 March 2016 [92].

The outbreak in Sierra Leone claimed the lives of 3956 persons and is believed to have infected 14 124 (8706 laboratory-confirmed cases) [47]. Sierra Leone is now the country with the largest number of Ebola cases in history.

### (d) Other countries affected

Several other neighbouring countries had confirmed Ebola infections during this period, all with epidemiological and genetically proven links to the outbreaks in Guinea, Liberia and Sierra Leone. However, only Nigeria and Mali had foci of local transmission. There was also importation of infections to several European countries and the USA, linked to the West African outbreaks, and in the USA and Spain isolated local transmission also occurred to HCWs. Table 3 highlights the Ebola infections diagnosed outside Guinea, Liberia and Sierra Leone by country. In addition, other countries also accepted evacuated cases from West Africa for hospitalization including Germany, France, Switzerland, The Netherlands and Norway. There was also the coincidental, but unrelated, outbreak in DRC which occurred at the same time.

**Table 3.** Cases diagnosed outside of West Africa related to this outbreak.

	no. cases	no. deaths	dates of outbreak	details
Nigeria [93]	20	8	23 July 2014–19 October 2014	index case travelled by air from Liberia. Local transmission to 19 people (12 first generation cases, 3 waves of transmission)total country case count (diagnosed and evacuated): 20
Mali [94–96]	8	6	24 October 2014–18 January 2015	two importations: 1. October 2014: 2-year old girl from Guinea whose father was a Red Cross worker who died—no local transmission 2. November 2014: Iman from Guinea, thought to have partaken in traditional burial ritual ceremonies across the border in Sierra Leone. Local transmission occurred and six others infected phylogenetic analysis confirms these were two separate introductions [97]total country case count (diagnosed and evacuated): 8
USA [98]	4	1	30 September 2014–21 December 2014	two episodes: 1. Liberian national visiting family in Dallas—local transmission to two HCWs 2. HCW returned from Guinea—no local transmission total country case count (diagnosed and evacuated): 4
Spain [99]	1	0	6 October 2014–2 December 2014	nurse caring for a repatriated HCW N.B. HCW diagnosed in Sierra Leone—local transmission occurred to nurse in Madrid. A second HCW was repatriated from Liberia, but no local transmission occurred total country case count (diagnosed and evacuated): 3
UK [100]	1	0	29 December 2014–10 March 2015	HCW returned from Sierra Leone (multiple hospital admissions for Ebola)—no local transmission N.B. two other cases were repatriated from Sierra Leone after diagnosis and cared for in UK—no local transmission total country case count (diagnosed and evacuated): 3
Senegal [101]	1	0	29 August 2014–17 October 2014	traveller from Guinea—no local transmission total country case count (diagnosed and evacuated): 1
Italy [102]	1	0	12 May 2015–20 July 2015	HCW returned from Sierra Leone—no local transmission total country case count (diagnosed and evacuated): 1

## 5. Outbreak: propagation and failure to control

Social, biological and structural drivers of transmission combined during the 2013-2016 outbreak to allow a perfect storm with unprecedented and devastating consequences [103]. This was exacerbated by a failure in the response at both national and international levels. Table 4 outlines the key factors leading to the failure in controlling the outbreak. This section discusses these factors in more detail, with specific focus on the role of interventions on limiting outbreak size.

**Table 4.** Factors leading to failure to control the outbreak.

factors	
population structure/geography	mobile populations rural to urban migration affecting densely populated areas zoonotic emergence event at the intersection of three countries and near a road network porous national borders (figure 4) multicountry spread
economic factors/lack of infrastructure	fragile states following recent civil wars lack of trust in government following historic corruption weak health systems road networks along which infection spreads poor transportation networks poor telecommunication networks international air links lack of vehicles to access remote sites
cultural and behavioural factors	traditional burial rituals dependence on traditional healers secret societies community resistance, fuelled by lack of trust and disregard to cultural sensitivities at times conspiracy theories, e.g. hiding cases civil disobedience
interventions/failure in response	delayed identification delayed and poorly coordinated international response weak governance and lack of local accountability within national/local response lack of evidence on effectiveness of interventions lack of experience in managing an outbreak on this scale lack of communication shortages of HCWs healthcare associated spread augmenting outbreak initial lack of community engagement and public information

### (a) Population structure/geography

**Figure 4.**
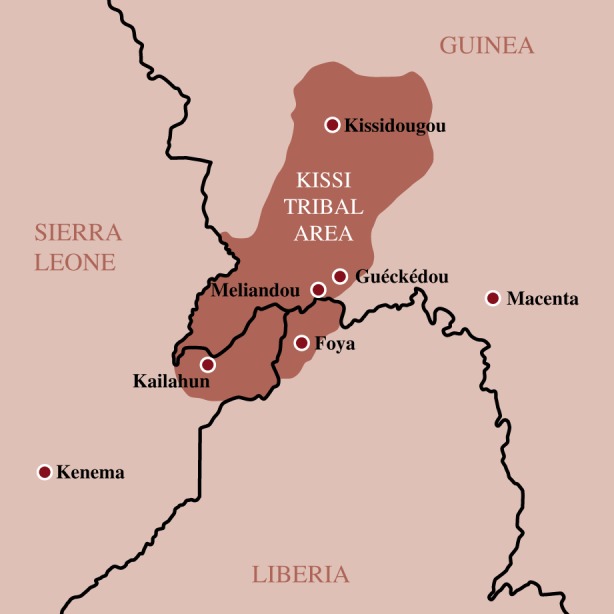
Where the outbreak began—map to show Kissi tribal area spanning Guinea, Sierra Leone and Liberia. (Online version in colour.)

Emerging diseases such as Ebola often arise from close animal contact at the zoonotic interface. Therefore, it is common for outbreaks to occur in isolated rural areas, and most previous Ebola outbreaks have remained contained in these settings. In the initial phases of this outbreak, disease transmission went undetected and likely led to chains of transmission within the Kissi tribal area that spans the borders of Guinea, Liberia and Sierra Leone (figure 4). The local population is mobile across these three countries, the borders of which are porous, and as a result it accounted for the vast majority of early cases in this outbreak.

Failure to control transmission in the early phases of the outbreak allowed mobile populations and migration to spread transmission chains from rural to urban areas. Recent studies estimate that population mobility in the major affected countries is seven times higher than elsewhere in the world, thought to be caused by poverty driving mobility as people look for work or food [104]. The increasing connectivity of distant rural communities [85] means that outbreaks of emerging diseases are more likely than ever to reach densely populated centres [105,106] such as Freetown, Monrovia, and Conakry. These major cities provide hubs for international spread [107–109] given that the world is increasingly globalized and infections do not respect national borders [109].

### (b) Lack of infrastructure

Guinea, Liberia and Sierra Leone are among the poorest countries in the world, and have only recently emerged from civil wars. Their damaged health infrastructure was ill-equipped to deal with the scale of this outbreak. Pre-outbreak, HCW capacity was already critically low at approximately one or two HCWs per 100 000 population (table 2), and this was further diminished by the epidemic [23,104].

The non-specific nature of EVD means fast and accurate laboratory diagnosis is essential, yet in rural West Africa both laboratory and human resource capacity was limited. In the initial stages this led to timely response mechanisms being hindered by a lack of diagnostic facilities. Furthermore, road systems, transportation and telecommunications networks were weak in all three countries, especially in rural settings. This delayed the transportation of patients and diagnostic samples and the dissemination of public information campaigns [104]. For example, in some settings, clinical samples had to be transported across large geographical areas with poor transport infrastructure, meaning diagnostic confirmation often took several days.

### (c) Cultural factors

#### (i) Burial practices

High-risk behaviours and lack of infection control measures around death and traditional burial practices have long been known to propagate transmission events [85]. During August 2014, 60% of new infections in Guinea were linked to funeral practices, with 80% linked to traditional burials in Sierra Leone in November 2014 [104]. One funeral alone is thought to have begun a huge chain of transmission with several hundred infections [110].

Safe burials were, therefore, integral to the Ebola response—modelling based on the outbreak in Liberia suggested that interrupting funeral transmission could have had the greatest potential impact of all interventions on outbreak prevention [111]. Despite this, repeated assessments revealed widespread risks in funerals including a lack of trained burial teams, a shortage of burial space, no clear guidelines on collecting diagnostic specimens from the deceased, and a lack of community engagement to facilitate culturally acceptable safe burials [112].

Funerals in eastern Sierra Leone, for example, are steeped in cultural significance; the strong sense of family and local allegiances, often tied together by marriages and dowries, are thought to have allowed these remote communities to survive through civil war when government safety nets were non-existent. The practice of taking wives from distant villages saw sisters and close female relatives travelling long distances in order to wash the body of women who died from Ebola according to the Muslim tradition (box 3), providing an important social pathway that facilitates the spread of Ebola to new geographical areas [85]. This was compounded by widowers travelling back to their deceased wife's home village to complete any outstanding dowry payments though labour, providing further opportunity for onward disease transmission [85].

New standard operating procedures developed by WHO (box 3) were insufficient alone to successfully reduce risk behaviours during funerals; it was only when these social pathways were recognized, acknowledged and addressed that the number of safe and dignified burials met international guidelines and the epidemic curve began to fall [113].

### (d) Interventions

Prior to this outbreak, the mainstay of interventions to combat Ebola outbreaks were contact tracing and follow-up for exposed contacts, prompt treatment and isolation of suspected and confirmed cases, strict infection control, and safe burial, underpinned by a strong commitment to community engagement [114]. These measures continued to be effective during the 2013–2016 outbreak, and the epidemic confirmed knowledge and protocols established from previous outbreaks.

However, progress in understanding the exact benefit of each intervention is limited owing to a lack of evidence; the decline in cases across all countries coincided with simultaneous implementation of multiple interventions and disentangling the role of each requires further study. For example, many interventions were part of an ‘improved package’ in Ebola treatment, where treatment beds and improved community-based infection control were implemented in tandem [115].

#### (i) Community engagement

Central to all interventions was the need to work with affected communities not only to effectively serve their needs [116], but also to enable the development and implementation of culturally acceptable strategies. As described throughout this paper, a failure to engage communities early enough had a detrimental effect on this outbreak response, but when effectively engaged, community interventions played a significant role in curtailing the outbreak. The three affected West African countries have cultures of strong community connectedness, with trust for community leaders far greater than that of government [117], exacerbated by recent civil unrest. Acknowledging these social structures and working alongside community networks proved essential for effective outbreak response.

Mistrust between the implementing organizations and individuals directly impacted the effectiveness of surveillance, contact tracing, healthcare seeking behaviour, and safe burial initiatives; all individually propagating the spread of the virus [118,119]. Conversely, where achieved, community engagement with strong international support was integral to controlling Ebola; modelling of the outbreak in Liberia concluded that the increase in bed capacity, credited with causing a reduction in disease incidence, was insufficient to bring case numbers down without significant public engagement [120].

Drawing on conversations with communities in the most affected countries, local experts and international actors, the main steps identified in achieving community engagement in Ebola response efforts include: identifying both male and female community leaders to champion key messages; organizing regular community meetings; using varied communication methods; tailoring global policies to local settings; and involving family members in care actions which do not expose them to increased risk [121].

Effective community engagement benefited policy-making: strategies designed when incorporating cultural values, customs and concerns of affected communities were more effective [121]. Implementation also benefited: at various stages of this outbreak, transmission was fuelled by a reluctance of populations to seek care in designated facilities, to engage in adequate contact tracing, to respect quarantine regulations, or to reveal deaths in order to allow safe burial [104]—all of which improved after programmes of community engagement [122]. Examples of successful interventions included communities buying megaphones to counter myths related to infection and to encourage people to seek treatment instead of hiding from authorities [59].

All other policies outlined below must, therefore, be viewed not as isolated technical interventions, but as part of a wider programme of disease control activities, with community engagement chief among these.

#### (ii) Contact tracing

The ability to identify, and subsequently interrupt, chains of transmission is crucial to the success of containment efforts, and the success of contact tracing is, like all Ebola interventions, determined by the extent to which communities trust and give accurate information to those attempting to curb the outbreak [123].

One proxy indicator as to the success of contact tracing is the proportion of new confirmed cases who were already being monitored as contacts of known, existing Ebola cases. These contacts can then be followed up with temperature checks for the 21 day incubation period and receive prompt isolation if diagnosed with Ebola, thereby improving treatment outcomes and reducing exposure to further potential contacts. In fact, modelling from early in the outbreak in Sierra Leone and Liberia identified that contact tracing (with prompt isolation and infection control) could have a more substantive effect on the epidemic than even potentially curative medical therapies [124]. Despite this, evidence from Guinea and Sierra Leone suggested that contact tracing was far from adequate at this time—as a result, few new cases were from identified contacts and chains of transmission proved stubbornly difficult to interrupt [119,125]. Alternative approaches focusing on community-based early detection over contact tracing proved more effective in simulations based on epidemic data [126]. In contrast, the rapid public health response to the various ‘tail end’ epidemics across the region involved effective contact tracing and a high proportion of secondary cases had been identified and followed up as contacts prior to their diagnosis with EVD.

#### (iii) Infection prevention and control measures

Effective infection control in healthcare facilities is a crucial step in interrupting chains of transmission. Lack of knowledge and resources to provide effective infection control were major factors leading to an amplification of this outbreak. Transmission was propagated by HCWs who became infected and who inadvertently spread infection to their family members and communities, particularly in the early phase of this outbreak [30], in a similar pattern to historic outbreaks [2,8,30]. Early in the outbreak, the relative risk for acquiring EVD was around 100 times higher for HCWs compared with the general population [127], although this risk decreased as barrier precautions were more effectively implemented, and personal protective equipment became available [128]. This is a tragic reminder of the risks frontline HCWs face in weakened and understaffed healthcare systems.

In an effort to strengthen infection prevention and control (IPC) practices, a partnership between Ministries of Health, CDC, WHO and others was established in August 2014 to improve IPC at non-ETC healthcare facilities and to decrease the risk for Ebola transmission to HCWs. National IPC plans were developed and published in each country by late 2014, with IPC task forces established to coordinate infection control efforts. However, progress was slow. For example, in October 2014, in Sierra Leone, almost five months after the first reported case and despite infection control teams being deployed, major gaps in IPC practices remained. None of the six Sierra Leonean districts visited by a CDC-led monitoring team had standard operating procedures or adequate equipment [129]. Progress was hindered by delays in importing personal protective equipment and a lack of engagement with community partners [121]. Thus, effective implementation on the ground was impossible despite extensive training [130].

#### (iv) Ebola treatment centres and bed capacity

The lack of bed capacity in ETCs to isolate and treat patients resulted in a massive inpouring of support to increase treatment facilities. Offering patients care with stringent infection control, supported by accurate PCR diagnosis, increased willingness to be hospitalized and facilitated contact tracing. ETCs have been integral in the control of previous outbreaks, though never before have they been deployed on this scale [131]. However, the role of increasing bed capacity in controlling the outbreak remains controversial. Modelling from the CDC in September 2014 indicated that 70% of all Ebola cases would need treatment in ETCs to begin to curb the outbreak [132], necessitating a huge scale-up in the provision of ETCs from the international community [133]. MSF had been operating several of the early ETCs, and several other players began to take an active role in providing more ETCs across all three countries.

The UN Mission for Ebola Emergency Response set a target of two ETC beds per suspected or confirmed case. This target was eventually met in January 2015 [134], but by this time the peak of the outbreak had passed and case numbers were already on the decline in most areas across the three countries. Despite this, several reports suggest that this intervention was perhaps the most crucial in controlling the outbreak [115,135], whereas others acknowledge that it occurred too late in the outbreak evolution to have significant impact [115,136]. Part of this discrepancy may be explained by the assumption in certain mathematical models that admission to ETCs led to near-perfect isolation of infected individuals [137]. Furthermore, constructing an ETC and training the relevant staff is a timely process, at a crucial time when a single week can make a big difference to the disease burden. Therefore, future outbreak response should begin construction of required ETCs early to maximize impact.

#### (v) Quarantine

Some interventions though appeared to be ineffective and sometimes even counterproductive. Epidemiological modelling provided little evidence to support the use of mass quarantines [138] by incorporating the evidence that when patients are asymptomatic they are not infectious [81]. For example, modelling after the Liberian West Point quarantine suggested it had little effect in bringing down the basic reproductive number (R0) [111]. Additionally, the quarantine of HCWs from high-income countries, as established by New York and New Jersey, was done with the aim to reduce risk, but its major impact appears to have been to dissuade other volunteers from travelling to West Africa [81].

#### (vi) Intervention summary

Given the limited resources, efforts were rightly focused on outbreak response and clinical care, rather than scientific advancement and research. As a result, we continue to lack sufficient data to inform a robust, evidence-based approach to control Ebola outbreaks and to determine how, when and in what order to deploy interventions. Prospectively collected data on outbreak response is sparse, preventing rigorous evaluation of the impact of the response on both incidence and the relative merits of each intervention [115]. The assessment of specific interventions was further limited by the contemporaneous introduction of many of the major strategies. It is likely that no single intervention was responsible for reversing the epidemic curve [136], but en masse the traditional control measures instituted appeared ultimately to help.

The limited public health infrastructures within the affected countries contributed to the failure to implement traditional public health measures early enough to deal with the scale of the outbreak. The shortcomings of these responses were compounded by the lack of cultural and contextual knowledge among the high-level international response. It appears that one of the key factors in controlling the outbreak was the successful engagement of communities to help overcome cultural barriers and to pass on educational messages about the importance of infection reporting, contact tracing and safe burial. Once communities understood that they were able to contribute to the response, successful implementation of all interventions increased.

Most of the progress in knowledge regarding successful interventions occurred via a process of elimination, i.e. by excluding interventions that did not play a crucial role in curbing case numbers based on timing of implementation (ETCs, vaccines and therapeutics). The proven interventions—exhaustive contact tracing, strict infection control measures (during burials and in healthcare settings) and community engagement—are not novel strategies. All were established during the response to the first Ebola outbreak of 1976 and they remain the mainstay of Ebola control today [116,120,121,139,140]. These traditional public health measures remain critical and need to be communicated and reinforced early on during future epidemics, especially in resource-poor settings. The response to this outbreak failed not in the development or deployment of novel technologies, but in the prompt recognition by global organizations of the scale of the outbreak. Box 4 suggests 10 components of an effective Ebola response based on our research for this article. They are in no particular order, and incorporate both practical solutions for which some evidence exists and recommendations from expert bodies.

Box 4. **Top 10 components of an effective Ebola response.**

1. Early identification and recognition of outbreak.
2. Effective collaboration and coordination between national and international players, with sound governance.
3. Quick mobilization of professional and community resources.
4. Improved communication and awareness.
5. Improved community engagement.
6. Training of HCWs in infection control.
7. Organization of contact tracing and isolation.
8. Good surveillance and case detection.
9. Safe burial practices.
10. Consideration of vaccination strategies based on the latest evidence.

### (e) Failure of response leadership

There has been much speculation and criticism regarding the timing and delay of both the national and international responses, with WHO in particular singled out for criticism [52]. This crisis exposed organizational failings in the functioning of WHO leading to a ‘significant and unjustifiable delay occurring in the declaration of a Public Health Emergency of International Concern’ [141]. The WHO has already accepted the need for transformation of organizational culture and delivery, but this will not be enough to prevent and mitigate future outbreaks—Member States must also transform to ensure full political support for implementing the core capacities for public health outlined in the International Health Regulations (IHRs; see below).

The scale of the outbreak was underestimated by experts and minimized by authorities. Although some key international players were quick to respond, the early phase of the outbreak was marred by resources being quickly overwhelmed and the inability of local public health infrastructure to cope with the rapidly amplifying case load. Guinea, Liberia and Sierra Leone had a shortage of public health surveillance capacities to detect, report and respond rapidly to the outbreak. The response was hampered by a lack of trained and experienced personnel willing to be deployed to West Africa, inadequate financial resources, a limited understanding of effective response methods, ineffective community engagement and poor coordination [141]. Furthermore, many suspect that the lack of good governance and accountability at a national/local level was a key factor in the genesis of the perfect storm that was the 2013–2016 Ebola outbreak.

## 6. Further questions

In the writing of this paper, many crucial questions were identified which remain the unexamined part of the story. These include: how might one account for the different epidemiological curves, geographical distributions, and mortality rates across the three countries?; why did the outbreak not spread more widely across porous borders to other neighbouring countries?; what is the role of protective immunity in controlling the outbreak?; is there any evidence for host genetic susceptibility to infection?; would the outbreak have died out without any intervention? This paper summarizes what is known and hence we have not speculated on any of these matters. However, we would like to highlight how much more work there is to complete before the outbreak can fully be understood.

## 7. Key successes

Overall, progress in our knowledge of EVD transmission dynamics, control measures and management strategies has been mixed. The limited research that did occur during the outbreak was focused on novel solutions, e.g. vaccines and therapeutics, rather than fully understanding traditional public health measures or clinical management (e.g. fluid and electrolyte balance). Areas of significant advancement include: establishing a pipeline for clinical therapeutic trials in emergency settings; vaccine and therapeutics development; the incorporation of near real-time molecular techniques into transmission dynamic studies; and the use of modelling to assist in decision-making.

### (a) Scientific progress

#### (i) Vaccine and therapeutics pipeline

Development of a process or ‘pipeline’, for rapid approval and implementation of clinical trials in emergency settings: this was one of the greatest advancements brought about by this outbreak—namely in relation to vaccines and therapeutics. The expedited ethical and regulatory approvals meant that patients could benefit from the latest scientific treatments without any meaningful delay. Although these vaccines/treatments lack the robust evidence base that would normally be required and hence are accompanied by potential risks, their use is justified in situations with significant associated mortality and limited opportunities to study the disease. This provides the possibility of being able to provide potentially life-saving treatments to some patients and to improve knowledge of disease management for future outbreaks.

The clinical trials occurred too late to have any significant impact on this outbreak, although they hold great promise for the future. Vaccine development in particular has been shown to be highly effective and safe [142–144] and will undoubtedly be used as a key preventative strategy in the future.

#### (ii) Molecular epidemiology

The use of cutting-edge molecular techniques: this was used in two main ways. First, rapid PCR was made available for the prompt and accurate diagnosis of suspected cases, facilitating their early isolation. Second, this outbreak was one of the first instances of real-time sequencing being used in an acute setting to enhance outbreak understanding. For example:

— Real-time genetic sequencing helped to identify specific clusters that could then be linked to risk behaviours. The large outbreak related to the funeral in Kenema, for example, was mapped after sequencing the virus in infected patients, and allowed future responses to be targeted to high-risk ‘super-spreading’ events [64]. Only when chains of transmission were identified, using a combination of conventional traditional field epidemiology and, later, cutting-edge, near real-time molecular techniques, were those infectious networks interrupted.
— Genomic surveillance also helped to describe the outbreak by facilitating an understanding of the origins of the outbreak and subsequent emergent clusters. Further, phylogenetic trees constructed during and after the outbreak demonstrated that disease transmission was almost exclusively within country, human-to-human transmission, rather than repeated zoonotic events [64].
— Genetic sequencing also established that the Ebola outbreak in the Democratic Republic of Congo in 2014 was a result of a separate, unrelated zoonotic event [145].

Therefore, novel technologies can provide additional benefit when used in tandem to supplement established strategies. This highlights that there is a role for incorporating viral genetic sequence data into the standard outbreak response toolkit, as it allows epidemiologists responding to the outbreak to focus their resources most effectively to interrupt transmission.

#### (iii) Mathematical models

The use of epidemiological models to assist complex decision-making: a large range of models were developed during the outbreak (described above) to inform decisions such as: where to allocate scarce resources in real time [146]; how to define the likely scope of the outbreak in the absence of control measures [124]; how to describe ‘worst-case scenarios' that stimulated international organizations and national governments to provide the necessary support; and how to help differentiate between effective interventions and those with limited utility [147]. A systematic review of mathematical models of the West Africa Ebola outbreak has made recommendations for further improvement of modelling in future outbreaks, including a degree of standardization of techniques to enable comparisons that could then be shared rapidly [148].

### (b) Clinical aspects

Many of the clinical and research findings from the 2013–2016 outbreak have helped confirm our prior knowledge of EVD [111], but progress has been limited. The data confirm previously documented incubation periods and the fact that people are not infectious until after they become febrile, giving additional support to the value of contact tracing, quarantine with fever surveillance and early isolation for possible and suspect cases [81].

Encouragingly, mortality rates did appear to decline throughout the outbreak [149]. Nonetheless, we acknowledge that this is subject to ascertainment bias of underreporting of milder cases early in the outbreak, and the difficulty in calculating a crude fatality rate from the proportion of fatal cases in the midst of the outbreak when the lag time between case reporting and case recovery or death alters the results [150]. It is possible that improved survival rates increased confidence in the healthcare system, leading to increased treatment seeking and better contact tracing, thus chains of transmission were more rapidly identified and disrupted.

### (c) Operational aspects

Finally, one of the most impressive aspects of the outbreak response was the unprecedented levels of international collaboration and cooperation. The response was truly global and included a diverse range of organizations and national delegations from across the world, including NGO, humanitarian, military and governmental bodies. The global health community should be indebted to all those who were involved in this response, but especially to NGOs, including MSF which first raised the alarm about the scale of this Ebola outbreak, and a host of others that were integral in establishing and maintaining an effective response.

## 8. Lessons learned

The response to the 2013–2016 Ebola outbreak in West Africa has been the subject of numerous independent assessments with four different panels convened to review the international response, identify failures in the response and provide recommendations to strengthen future responses (box 5). All four panels concluded that the slow response of the WHO was a significant factor in the outbreak not being contained. Recommendations common to all include enhanced global collaboration during outbreaks including global governance and leadership, and the development of strong national core capacity in public health across the globe, as agreed under the IHRs [114]. The IHRs are negotiated global agreements for stronger health security and require all countries to develop and sustain eight core capacities in public health in order to better detect and respond to hazards, including infectious disease outbreaks. They also mandate international cooperation as a safety net when outbreaks spread across international borders, and pursuant to the Ebola outbreak, an international committee was convened to assess the effectiveness of the IHR.

Box 5. **Summary of common recommendations from the four independent assessment panels.**

**• Independent assessment panels**

— *WHO Ebola Interim Assessment Panel* [141]
— *Harvard University and the London School of Hygiene and Tropical Medicine's Independent Panel on the Global Response to Ebola* [52]
— US National Academy of Medicine's *Commission on a Global Health Risk Framework for the Future* [151]
— United Nations High-Level Panel on the *Global Response to Health Crises* [152].

**• Recommendations**

Preventing and responding to outbreaks

WHO's role – WHO to:

— Re-establish leadership role as the guardian of global public health:
  (i) WHO to lead outbreak response with Member States supporting WHO at the frontline by implementing core capabilities in public health (surveillance, issuing alerts, and outbreak responses)
— Scale back its broad remit and refocus on providing technical support
— Create a dedicated ‘Centre for Emergency Preparedness and Response’ with strong technical expertise
— Create a Standing Emergency Committee with strengthened ability to independently identify health risks and declare public health emergencies appropriately
— Ensure a protected budget and contingency fund to support this new centre and allow rapid deployment of emergency response when required; this would include annual contributions from Member States, International Monetary Fund, World Bank, and other multilateral donors
— Develop a plan to ensure core capacities required under IHR for all countries (with World Bank) including:
  (i) strategies to ensure that governments invest in building core capacities to detect, report and respond rapidly to outbreaks
  (ii) new approach to staffing WHO country offices, ensuring the highest-level capacity for vulnerable countries (with weak health systems and governance challenges)
— WHO and partners to ensure that appropriate community engagement is a core function of future health emergency responses.
— Governance reforms to rebuild trust in WHO.

International Health Regulations (IHR) - IHR review committee to:

— Consider the possibility of an intermediate level alert to engage the international community at an early stage without declaring a full Public Health Emergency of International Concern
— Consider incentives to encourage countries to notify public health risks to WHO (e.g. public commendations and/or financing mechanisms to mitigate adverse economic effects), and disincentives to discourage countries from instigating travel restrictions and limit trading without scientific justification or WHO recommendation.

Other mechanisms:

— Clear response mechanisms for coordination and escalation in health crisis, including mobilization of the UN system for humanitarian crises, and cooperation with non-state actors including civil society organizations, the private sector and the media
— Consideration of formalized regional and sub-regional level arrangements to enhance prevention of and response to health crises.

*Research: production and sharing of data, knowledge, and technology*

— Develop a framework for research and development operations in outbreak settings to enable, accelerate and ensure good governance. Timely roll out of successful results should also be encouraged. WHO should play a central convening role
— Develop a worldwide financing facility for outbreak-relevant diagnostics, vaccines, therapeutics, and medical and information technology (with no commercial incentives).

*Governing the global system for preventing and responding to outbreaks*

— Create an independent UN Accountability Commission to oversee the new response centre, monitor compliance with the IHR's Core Capacity requirements at a country level, and assess function in outbreak responses
— Create a UN Security Council led Global Health Committee to put global health issues at the centre of the global security agenda and expedite high-level leadership
— WHO Executive Board should mandate good governance reforms at the country level, including establishing a freedom of information policy. They should have the capacity to challenge governments and hold them publically accountable for protecting public health.

It is perhaps surprising to note that many of the recommendations suggested by these four panels and other experts mirror the main conclusions from Heymann *et al.* after the Kikwit outbreak over 20 years ago [153]. These include the need for a stronger infectious disease surveillance (both national and global); improved international preparedness to provide support when similar outbreaks occur; coordinated Ebola research (especially for valid diagnostic tests), better patient management procedures and identification of the natural reservoir. We must hope that after an epidemic of this scale, these recommendations will now be heeded and enacted to improve global health security going forward.

## 9. Conclusion

The 2013–2016 Ebola outbreak in West Africa was described by the WHO as an ‘old disease in a new context’ [104] owing to the many unique aspects it displayed: it was the largest Ebola outbreak in history, being larger than all others combined; it was the longest in duration; it was the first with multicontinental spread, initially within Africa and then to Europe and North America; and it was the first in which there was a pipeline of clinical products that could be studied for effectiveness.

Although this outbreak was quantitatively many times larger than previous outbreaks, it was not qualitatively different, as the means of amplification and transmission were the same as previously described. As a result, the control measures designed and implemented in prior outbreaks were crucial to the response, but the failure to implement these in a timely manner had devastating consequences. Therefore, it is clear that in future epidemics these old messages need reinforcing early and vocally.

In addition, this outbreak highlighted the crucial role of early community engagement in implementing a successful response. Moreover, although previously established measures will remain the mainstay of control strategies, we learned during this outbreak that novel technologies (e.g. real-time sequencing) can provide additional benefit when used in tandem to supplement established strategies.

The scale and duration of this outbreak provided a unique opportunity to develop and test evidence-based protocols to improve both basic supportive clinical management (e.g. fluid and electrolyte treatments) and the implementation of public health interventions. However, woefully little progress was made in these areas and the same questions remain pertinent now as were raised after the Kikwit outbreak over 20 years ago [153]. Had the key lessons from historical outbreaks been acted upon, thousands of lives could have been saved during the 2013–2016 outbreak. In addition, this failure resulted in a lack of scientific progress which in turn means an opportunity has been missed to improve outcomes of future outbreaks. This is, perhaps, one of the biggest failings in the response. One can only hope that we have now learned the lesson, and that the global community will both heed the recommendations suggested and translate these into practical solutions to make a difference going forward.

Although the initial outbreak response was slow and inadequate, once established the response demonstrated unprecedented and impressive levels of international cooperation [154]. National governments and the global health community should be indebted to all the organizations and individuals who took great personal risk and showed courage in contributing to the effective response. This outbreak was also a salutary lesson: collaborations and structures are in place and must now be better used to enable a rapid and effective national response to outbreaks, while also providing a global safety net for response when national efforts fail to prevent international spread. Further, there is evidence that the IHR are working better: the Emergency Committee of the IHR recommended to WHO to declare the outbreak of Zika virus a global public health emergency of international concern in February 2016 [155].

While at the time of writing the 2013–2016 outbreak of Ebola in West Africa has been declared over, WHO continues to stress that ‘Sierra Leone, Liberia and Guinea are still at risk of Ebola flare-ups, largely due to virus persistence in some survivors, and must remain on high alert and ready to respond’ [156].

## Endnotes

1 To avoid confusion, it is important to note that all eight prefectures in Guinea have a district and city (the regional capital) with the same name, therefore we have tried to be explicit in stating the prefecture, district, and town level within this paper.

2 The International Red Cross, Samaritan's Purse, Pentecostal Mission Unlimited-Liberia, CHF-WASH Liberia, PLAN-Liberia, UN FPA.
